# *Triteleia peyerimhoffi* comb. n., a remarkably variable circum-Mediterranean scelionid (Hymenoptera, Platygastroidea)

**DOI:** 10.3897/zookeys.140.1925

**Published:** 2011-10-26

**Authors:** Ovidiu Alin Popovici, Ferdinando Bin, Lubomir Masner, Mariana Popovici, David Notton

**Affiliations:** 1 University ‘Al. I. Cuza’ Iasi, Faculty of Biology, B-dul Carol I, no. 11, RO – 700506; Romania; 2 Department of Arboriculture & Plant Protection, Entomology, University of Perugia, 06121, Perugia; 3 Agriculture & Agri-Food Canada, Ottawa, Ontario K1A 0C6, Canada; 4 Department of Entomology, The Natural History Museum, Cromwell Road, London, SW7 5BD, United Kingdom

**Keywords:** Hymenoptera, Platygastroidea, microhymenoptera, egg parasitoids, *Caloteleia peyerimhoffi*, *Triteleia dubia*, variability

## Abstract

*Triteleia peyerimhoffi* **comb. n**. (Kieffer, 1906) is redescribed taking into account its great variability and is considered the senior synonym of *Triteleia dubia* (Kieffer, 1908), *Calliscelio lugens* (Kieffer, 1910) and *Triteleia striolata* Kononova & Petrov, 2000, **syn. n.** Neotypes are designated for *Triteleia dubia* and *Triteleia peyerimhoffi*. *Triteleia peyerimhoffi* is a new record for Greece, France and Croatia and was reared for the first time from eggs of Orthoptera laid in the dead wood of *Quercus* sp. and *Tilia* sp. in Romania.

## Introduction

Jean-Jacques Kieffer (b. 1857 – d. 1925) was an Abbé, a clergyman, and taught natural history and religion at the Collége Saint-Austin at Bitche in Lorraine (Nominé 1926). His taxonomic work was published in a large number of scientific papers and in some comprehensive monographs, e.g. *Das Tierreich* and André’s *Hyménoptères d’Europe et d’Algérie*. During his life, he described 49 genera and 465 species belonging to the Platygastroidea (Johnson 1992). Kieffer had a private collection, the remnants of which are at the Muséum national d’Histoire naturelle in Paris (Notton 2004). He also published on much material belonging to other collectors and museums, and this was returned to them, so Kieffer’s types are scattered in collections around the world and many types have yet to be found. Hence, many species described by Kieffer have an uncertain status today.

*Triteleia peyerimhoffi* was described by Kieffer (1906) under the name *Caloteleia peyerimhoffi* (Kieffer interpreted *Calotelea* following Ashmead rather than Walker). The type material, all female, was obtained from eggs of *Ephippiger confusus* (now *Uromenus brevicollis* (Fischer) according to Sahnoun et al. (2010) from Algeria by Dr. Paul Peyerimhoff. Peyerimhoff (1908) published a paper about the biology of *Ephippiger confusus* and mentioned *Caloteleia peyerimhoffi,* including some notes about its oviposition behaviour.

In 1908, Kieffer, described *Ceratoteleia,* including species with a characteristic long metasoma with a horn on the first metasomal tergite, and with the length of the marginal vein varying from punctiform to the same length as the stigmal vein. Two years later (Kieffer 1910a), transferred *Caloteleia peyerimhoffi* to *Ceratoteleia*. In this genus Kieffer (1910b) described two more new species similar to *Caloteleia peyerimhoffi*: *Ceratoteleia lugens* from France and *Ceratoteleia mediterranea* from Italy. *Ceratoteleia* Kieffer is in fact the same as *Caloteleia* sensu Ashmead (Ashmead’s unjustified emendation of *Calotelea* Westwood).

Kieffer obviously did not study Ashmead’s type of *Caloteleia grenadensis* (in BMNH) and therefore his concept of *Ceratoteleia* is very heterogeneous, containing species of several genera (e.g. *Triteleia*, *Calliscelio*, *Holoteleia*, *Probaryconus*, etc.). Masner (1976) synonymized *Ceratoteleia* under *Calliscelio* Ashmead.

Silvestri (1939) described the biology of *Uromenus* and gave some information about its parasitoids. He showed a picture of some parasitoids ovipositing into eggs of *Uromenus*, identified as *Ceratoleia* (a misspelling of *Ceratoteleia*). Petit et al. (2007) have some excellent pictures of *Uromenus brevicollis* and scelionids ovipositing into its eggs. They identified these as *Catoteleia peyerimhoffi* (a misspelling for *Caloteleia peyerimhoffi*).

*Triteleia dubia* was described by Kieffer (1908) in *Apegus*, subgenus *Parapegus* based on one male from Hungary. According to Kieffer, the subgenus *Parapegus* is distinct from the rest of *Apegus* because of the long marginal vein (almost equal with the stigmal in *Parapegus* and punctiform in other *Apegus*) and because of the sculpture of the head (foveolate in *Parapegus* and with striae in other *Apegus*). Kieffer (1908) divided all genera known to him without a dorso-ventrally flattened body and with the scutellum unarmed into two groups: firstly genera with three longitudinal grooves on the mesoscutum and secondly those with two or no longitudinal grooves on the mesoscutum. He included *Triteleia* and *Apegus* in the second group.

Two years later Kieffer (1910a) kept *Apegus* divided into two subgenera: *Apegus* and *Parapegus* on the basis of the same characters, but later he considered *Parapegus* as a distinct genus (Kieffer 1914, 1926).

Masner (1956) revised *Parapegus* and described a female allotype of *P*. *dubius* from one specimen from Moravia caught on 14 September 1936 by F. Gregor in grassland (specimen catalogue no. 3 109 – NMPC). Later Masner (1976) synonymized *Parapegus* with *Macroteleia* and transferred *Parapegus dubius* to *Triteleia*. This was correct because Masner recognised that the median longitudinal mesoscutal furrow in *Triteleia* is only a specific, not a generic character. *Parapegus dubius* was transferred to *Triteleia* because of the shape of T6 in the female, which is depressed dorsoventrally to form a flat triangle, and because T7 in the male is armed posterolaterally with two sharp spikes, or at least tiny points.

Kozlov (1978), probably without seeing Masner’s (1976) paper, described the genus *Parapegus* again, adapting Masner’s (1956) description and reusing drawings from the same paper. This was the only mention of this species in the Russian literature. (Kozlov and Kononova (1985, 1990), Kononova and Petrov (2000) and Kononova and Kozlov (2008) do not mention *Parapegus* *dubius* or *Triteleia* *dubia*. Conversely Johnson(1992) mentioned *Triteleia dubia* (Kieffer, 1908) as a valid Palaearctic species, Bin et al. (1995) recorded it from Italy, and Popovici (2005) recorded this species from Romania based on one female from the Bârnova forest (N.–E. Romania).

Our goal in this paper is to provide a modern description of this species, document its unusual variability and provide new data about its biology. The contributions of the authors are as follows: O.A. Popovici (character definition, species concept development, imaging, collection of new material, manuscript preparation); F. Bin (had the idea for the paper, provided most of the Italian material, and contributed to the section on biology); L. Masner (character definition, species concept development, provided new material from Italy, Hungary and France and elaborated the plan for this paper); M. Popovici (geometric morphometric analysis); David Notton (provided specimens from the BMNH and corrected the English of the paper). Any nomenclatural acts in this paper are to be attributed to O. A. Popovici and L. Masner.

## Material and methods

Most of specimens seen were caught with a Malaise trap in various parts of Europe, especially the south and west including: France, Greece, Hungary, Italy and Romania. One specimen each from Croatia and Romania were swept. The remaining specimens were reared from the dead wood of *Tilia* species and *Quercus* species. Specimens were glued to triangular card points. For better examination, the maxillo-labial complex, one antenna, legs and wings of some specimens were removed and mounted on microscope slides. Specimens were examined using a Kruss MSZ54 stereomicroscope. Microscope slides were analyzed with a Euromex GE 3045 microscope, and drawings were made using a Reichart drawing tube.

### Abbreviations and morphological terms used in text:

**A1, A2, ... A12**: antennomeres 1-12; **DPO**: diameter of posterior ocellus; **fmc**: foramen magnum capitis; **gen**: gena; **ha**: hypostomal area; **HE**: height of compound eye; **hf**: hypostomal folds; **Hfd**: height of frontal depression; **ihc**: inner hypostomal carina; **Lck**: length of central keel; **LE**: length of compound eye; **Lfw**: length of fore wing; **LH**: length of head (measured at level of anterior ocellus); **Lhw**: length of hind wing; **LOL**: lateral ocellar line, the shortest distance between inner margins of anterior and posterior ocellus; **Lscut**: length of scutellum; **Lt**: length of temple; **MLC**: maxillo-labial complex; **ocp**: occiput; **ocpc**: occipital carina; **ohc**: outer hypostomal carina; **OOL**: ocellar ocular line, the shortest distance from inner orbit of compound eye to the outer margin of lateral ocellus; **pg**: postgena; **pgb**: postgenal bridge; **POL**: posterior ocellar line, the shortest distance between inner margins of posterior ocelli; **sgp**: sub-genal process; **T1, T2, ... T6**: metasomal terga 1-6; **tb**: tentorial bridge; **Wfw**: maximum width of fore wing (measured perpendicular to fore wing margin); **WH**: maximum width of head; **Whw**: width of hind wing; **Wscut**: width of scutellum.

**Morphometric analysis**. In total 82 specimens of *Triteleia* were measured. 60 females: Croatia (1); France (18); Greece (7); Hungary (3); Italy (27); and Romania (4), and 22 males: Greece (3); and Italy (19).All measurements were made using a Kruss MSZ54 stereomicroscope at 90× magnification.

The following characters were measured: body length; LH; WH; POL; LOL; DPO; HE; LE; distance between compound eyes (measured at level of anterior ocellus); Lt; distance between toruli; Lck; Hfd; surface of frontal depression covered with transversal striae; distance between compound eye and frontal depression; length of cheek; length and width for all antennal segments (A1….A12); length of mesosoma; width of mesosoma; length of mesoscutum; Lscut; Wscut; length of metascutellum; width of metascutellum; distance between lateral propodeal carina; width of lateral propodeal area; Lfw; Wfw; length of marginal vein; length of stigmal vein; length of postmarginal vein; Lhw; Whw; length of marginal fringe of hind wing (at level of hamuli); length of metasoma; length of T1; minimum width of T1; maximum width of T1; length of T2; maximum width of T2; length of T3; maximum width of T3; length of T4; minimum width of T4; length of T5; minimum width of T5; length of T6; minimum width of T6.

For each ratio in the description of species we used minimum – maximum (mean ± standard deviation).

The relationships between specimens were analyzed using Principal Component Analysis (PCA). This was performed using log-transformed data on a variance–covariance matrix (Klingenberg 1996). The Jolliffe cut-off value was used to indicate the number of significant principal components and standard errors of these were also determined with a bootstrap procedure (Boot N = 1000). We used the Kolmogorov-Smirnov test to show the distribution of metric data within all populations (all populations were normally distributed, p>0.05). The Levene test was used to test the homogeneity of the variance, a one-way ANOVA was conducted on the whole data set to test significant differences between variables and then a *post hoc* test defined pairwise differences in variables of populations (Sokal and Rohlf 1995). Depending on the result of the Levene test we used the Tukey or Games–Howell test.

The metasoma was analyzed using geometric morphometric methods based on the Kendall theory of shape (Kendall 1977). The shape is a configuration of Cartesian coordinates of landmarks which are discrete anatomical homologues (Zeldith et al. 2004). Generalized Procrustes Analysis (GPA) was performed to superimpose landmark configuration; it removes variation due to differences in translation, orientation, size and superimposes the objects in a common coordinate system. We generated thin-plate spline deformation grids to visualize metasomal shape differences. Size and shape components of this configuration were separately analyzed. The size of the metasoma was measured as a centroid size (CS). The CS is a geometric scale which is mathematically defined by the square root of the sum of squared distances between all landmarks and their centroid (Zelditch et al. 2004).We collected 21 homologous landmarks on the metasoma using tpsDig2 (Rohlf 2006). The overall metasomal size variation has been presented as a box plot, while differences between populations have been tested with a Pairwise Analysis of Variance (ANOVA) using post-hoc Tukey Least Square Distance test.

Relationships between metasoma shapes were investigated using Principal Component Analysis (PCA). PCA was performed on all shape variables in order to define the greatest axes of metasoma shape variation in the dataset. The visualization of the shape differences was made with thin-plate spline deformation grids. Overall differences between metasoma shapes have been tested by a Multivariate Analysis of Variance (MANOVA) and the permutation tests (1000 permutations) for Mahalanobis distances among populations were performed to confirm the significant differences. The effect of size on shape was investigated by multiple regression.

Statistical analysis was performed using Morpho J (Klingenberg 2008), tps software (Rohlf 2007), SPSS vers. 13 and PAST vers. 2.09 (Hammer et al. 2001).

Morphological terminology follows (Masner (1980, 1983) and Mikó et al. (2007). Terminology of surface sculpturing is from Harris (1979).

### Acronyms of collections:

CNCICanadian National Collection of Insects, Ottawa, Canada


OPPCO. Popovici personal collection, University ‘Al. I. Cuza’ Iasi, Romania


FBINCollection of F. Bin, Università di Perugia, Perugia, Italy


BMNHNatural History Museum, London, United Kingdom


MNHNMuséum national d’Histoire naturelle, Paris, France


HNHMHungarian Natural History Museum, Budapest, Hungary


## Results

### Triteleia peyerimhoffi

(Kieffer, 1906) comb. n.

http://species-id.net/wiki/Triteleia_peyerimhoffi

Caloteleia peyerimhoffi Kieffer 1906: 6; Peyerimhoff 1908: 515.
Apegus dubius Kieffer 1908: 151, 163. syn. n.
Apegus (*Parapegus*) *dubius *: Kieffer 1910a: 86.
Ceratoteleia peyerimhoffi : Kieffer 1910a: 89; Kieffer 1914: 321; Kieffer 1926: 501, 503.
Ceratoteleia lugens Kieffer 1910b: 310; Kieffer 1914: 317; Kieffer 1926: 501, 502. syn. n.
Parapegus dubius : Kieffer 1914: 310; Kieffer 1926: 497, 498; Masner 1956: 237; Kozlov 1978: 616; Cavalcaselle 1968: 319.
Triteleia dubia : Masner 1976: 29; Johnson 1992: 507; Bin et al. 1995: 15; Popovici 2005: 16.
Calliscelio peyerimhoffi : Johnson 1992: 359; Kononova and Kozlov 2008: 258, 262.
Calliscelio lugens : Johnson 1992: 358, Kononova and Kozlov 2008: 259, 266.
Triteleia striolata Kononova and Petrov 2000. syn. n.
Catoteleia peyerimhoffi (misspelling): Petit et al. 2007: 148.

#### Description.

Body size: female 3.0–4.6 mm (3.9 ± 0.4, n = 60); male 3.4–4.1 mm (3.6 ± 0.2, n = 22).

Colour: body black; antenna brown with reddish tint on some parts: radicle yellow with reddish tint; A1–5 with reddish tint on the ventral side; wing veins brown; legs light brown, sometime yellowish; middle of femora with dark tint.

**Figure 1.**
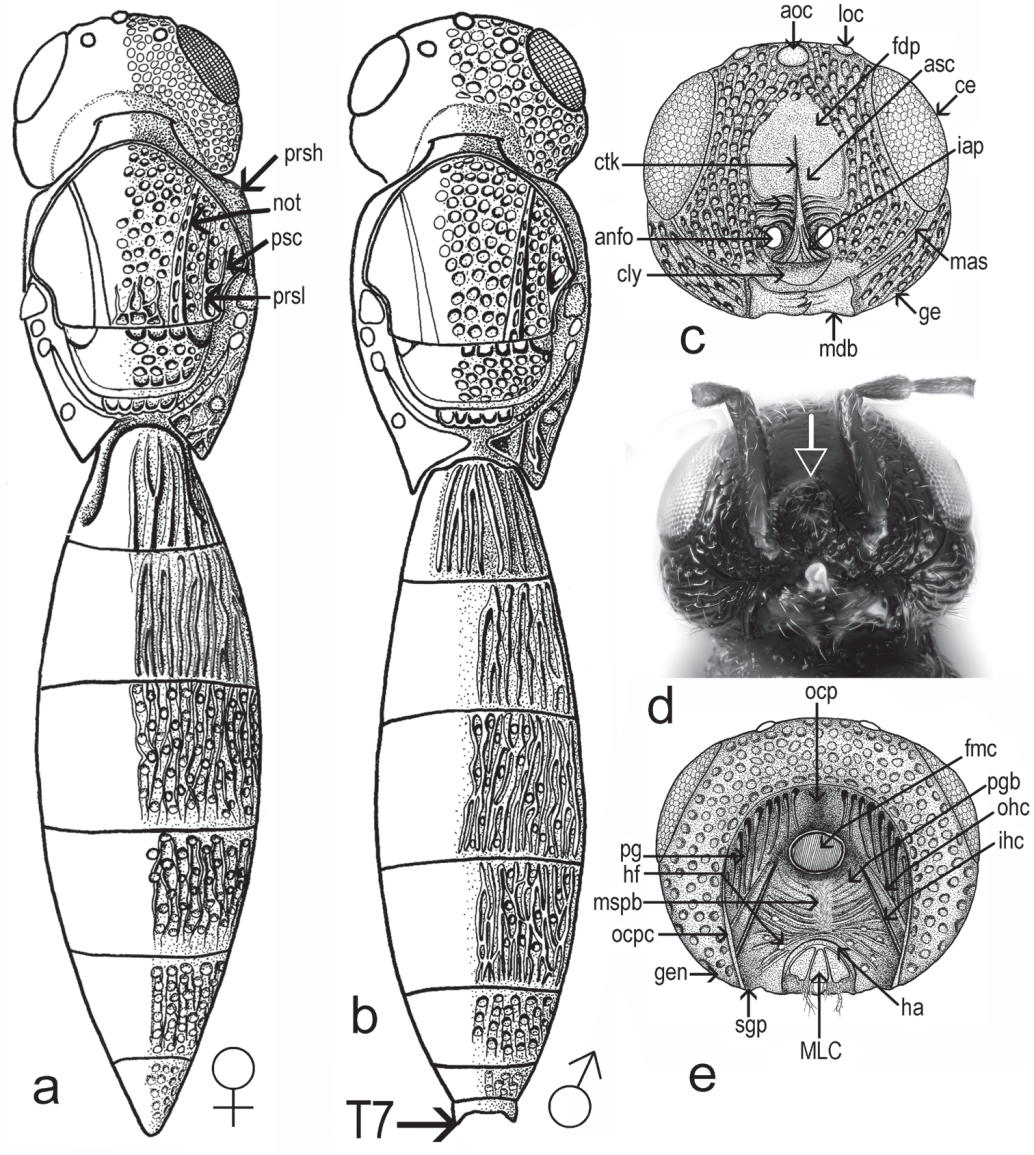
*Triteleia peyerimhoffi*: **a** – habitus female, dorsal view **b** – habitus male, dorsal view **c** – head, frontal view **d** – head, frontal view in malformed specimen **e** – back of head.

**Figure 2.**
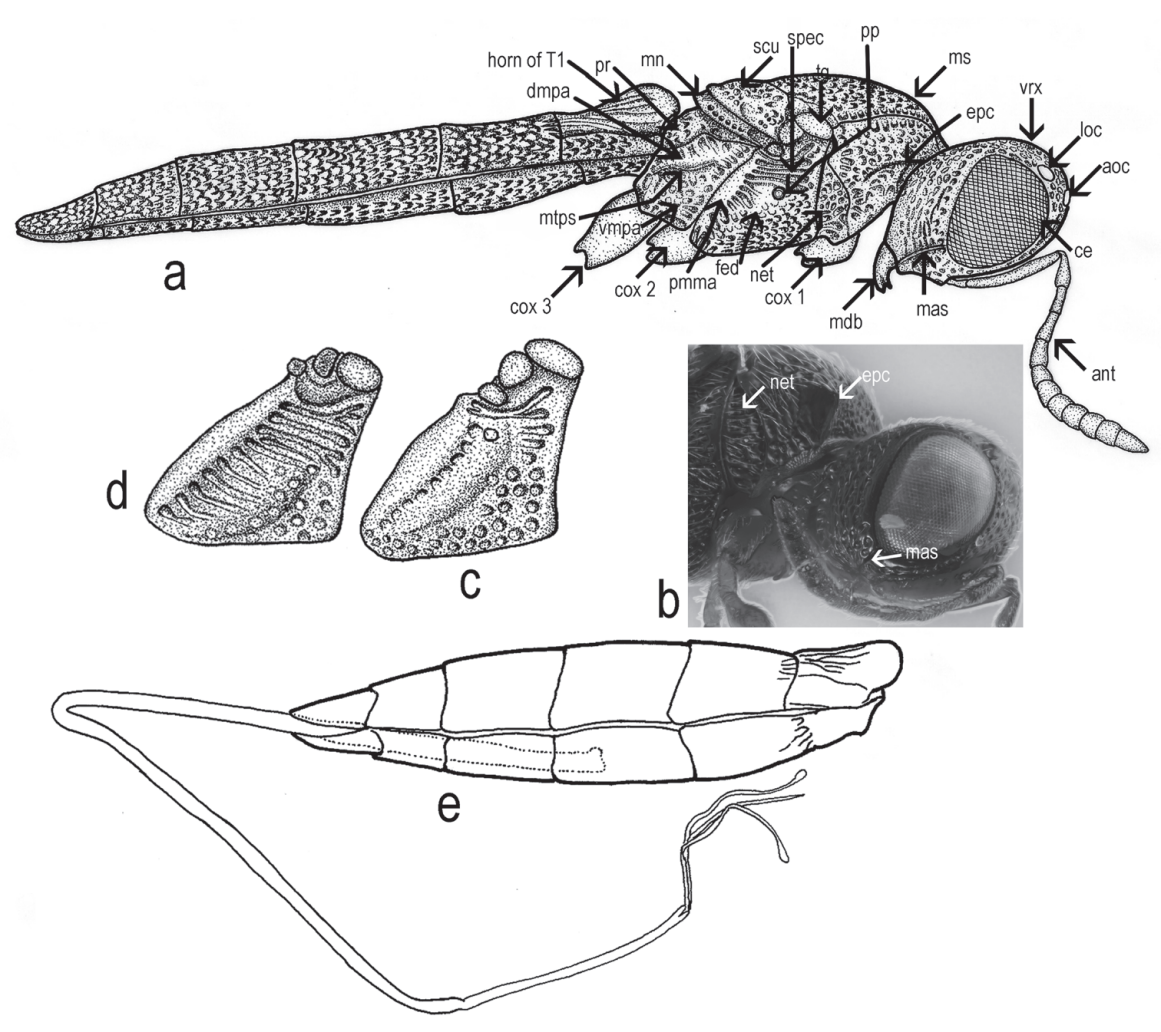
*Triteleia peyerimhoffi*: **a** – habitus, lateral view **b** – head and pronotum, lateral view **c** and **d** – variability of sculpture in mesopleuron **e** – metasoma and ovipositor system.

Head shape: dorsal view transverse, width 1.6–2.0 times length in female (1.8 ± 0.1, n = 60), 1.6–1.8 times length in male (1.7 ± 0.05, n = 22), 1.0–1.1 times width of mesosoma in female (1.02 ± 0.03, n = 60). Hyperoccipital carina absent. Occipital carina present, smooth, almost absent in median part**.** Compound eye large, glabrous. Eye width 1.6–2.8 times temple width in female (2.2± 0.3, n = 60), 1.5–2.3 times temple width in male (1.9 ± 0.2, n = 22) and 1.7–4.1 times distance between eye and frontal depression in female (3.1 ± 0.5, n = 60), 2.0–3.3 times distance between eye and frontal depression in male (2.5 ± 0.3, n = 22). Eye height 1.2–1.4 times width of eye in female (1.2 ± 0.05, n = 60), 0.8–1.0 times width of eye in male (0.9 ± 0.06, n = 22) and 1.6–3.2 times length of cheek in female (2.3 ± 0.25, n = 60), 1.9–2.4 times length of cheek in male (2.1 ± 0.1, n = 22). Inner orbits nearly parallel, diverging only in ventral half. Length of diameter of posterior ocellus 1.3–2.7 times OOL in female (2.0 ± 0.3, n= 60), 1.3–2.5 times OOL in male (2.2 ± 0.3, n = 22). POL 1.3–2.3 times LOL in female (1.7 ± 0.19, n= 60), 1.3–2.0 times LOL in male (1.7 ± 0.2, n = 22). Distance between compound eyes (measured at level of anterior ocellus) 1.5–2.1 times POL in female (1.8 ± 0.12, n = 60), 1.6–2.1 times POL in male (1.9 ± 0.09, n = 22). Orbital carina absent; frontal depression shallow, unmargined, submedian carina absent; antennal scrobe present, shining; central keel on frons (ctk Fig. 1c), present, not bifurcate, only a weak trace in some specimens. Length of central keel 0.2–0.9 (0.5 ± 0.2, n = 60) times height of frontal depression in female. Base of frontal depression transversely striate (Fig. 1c). The transverse striation is very variable 0.1–0.5 (0.3 ± 0.09, n = 60) times height of frontal depression in female. Interantennal prominence (iap Fig. 1c) moderately produced, torulus opening on antero-frontal surface of prominence (in one specimen, the interantennal prominence was hypertrophied, so that the distance between the toruli was twice than of normal specimens (Fig. 1d). Malar sulcus (mas, Figs 1c; 1d; 2a; 2b) present, fine, deeply incised, almost straight, running from lower margin of eye to mandibular articulation. Genal carina absent. Cheek without costae arising from anterior mandibular articulation. Clypeus (cly Fig. 1c) very small, narrow, semicircle, without corners produced laterally. Mandible strong, relatively short and broad, apex tridentate, teeth subequal in length, acute, ventral toothslightly longer. Number of maxillary palpomeres 4; labial palpomeres 2.

Sculpture of head (Figs 1a; 1b; 1c; 1d; 1e; 2a; 2b): vertex, interocellar space, cheek and space between compound eye and frontal depression foveolate. Frontal depression shining in apical half, transversely striate basally (Fig. 1c). In some specimens the lateral sides of frontal depression are longitudinally striate.

**Figure 3.**
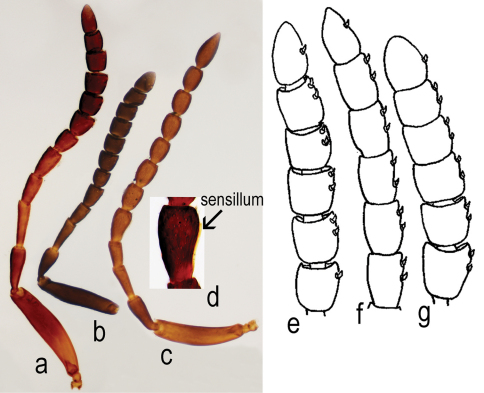
*Triteleia peyerimhoffi*: **a** – antenna in female from Italy **b** – antenna in the smallest specimen female **c** – antenna in male **d** – detail with 5 antennal segment, “sex – segment” **e** – clava in female of *Triteleia peyerimhoffi* **f** – clava in female of *Apegus* sp. **g** – clava in female of *Macroteleia* sp.

Antenna 12-segmented in both sexes (Figs 2a; 3a; 3b; 3c). Length of A1 4.0–6.25 times width in female (4.8 ± 0.38, n= 60), 4.0–4.6 times width in male (4.3 ± 0.2, n = 22), 2.0–2.8 times length of A2 in female (2.4 ± 0.18, n= 60), 2.1–2.6 times length of A2 in male (2.4 ± 0.13, n = 22). Length of A2 2.0–3.6 times width in female (2.5 ± 0.29, n= 60), 1.8–3.3 times width in male (2.2 ± 0.3, n = 22) and 0.7–1.57 times length of A3 in female (0.9 ± 0.14, n= 60), 0.9–1.1 times length of A3 in male (1.0 ± 0.07, n = 22). A3, in female, the longest funicular segment, 2.3–4.6 times width (3.4 ± 0.4, n= 60), 2.0–3.3 times width in male (2.5 ± 0.3, n = 22), 1.0–2.25 times length of A4 in female (1.4 ± 0.18, n= 60), 1.3–1.7 times length of A4 in male (1.5 ± 0.13, n = 22). Length of A4 1.3–3.6 times width in female (2.1 ± 0.4, n= 60), 1.25–2.0 times width in male (1.5 ± 0.2, n = 22) and 0.8–1.4 times length of A5 in female (1.0 ± 0.12, n= 60), 0.6–0.9 times length of A5 in male (0.8 ± 0.06, n = 22). Width of A4 0.6–1.0 times width of A5 in female (0.88 ± 0.1, n= 60), 0.7–1.0 times width of A5 in male (0.8 ± 0.06, n = 22). Length of A5 1.3–2.5 times width in female (1.8 ± 0.3, n= 60), 1.4–2.0 times width in male (1.7 ± 0.1, n = 22) and 1.0–1.75 times length of A6 in female (1.3 ± 0.15, n= 60), 1.1-1.5 times length of A6 in male (1.3 ± 0.08, n = 22). Length of A6 1.0–2.0 times width in female (1.2 ± 0.2, n= 60), 1.2–1.8 times width in male (1.4 ± 0.1, n = 22) and 0.8–1.4 times length of A7 in female (1 ± 0.14, n= 60), 0.9–1.0 times length of A7 in male (1.0 ± 0.01, n = 22). Clava in female non-abrupt; claval formula A7–12: 1:2:2:2:2:1, differing from claval formula of *Apegus* and *Macroteleia* (in both cases 2:2:2:2:2:1; (Figs 3e; 3f; 3g)). Male antenna non-clavate; A5 sexually modified (Figs 3c; 3d). Length of A12 1.0–1.75 times width in female (1.4 ± 0.02, n= 60), 1.8–2.5 times width in male (2.3 ± 0.3, n = 22) and 1.0–1.75 times length of A11 in female (1.2 ± 0.15, n= 60), 1.5–2.0 times length of A11 in male (1.7 ± 0.1, n = 22).

Back of head (Fig. 1e): occipital carina present, with vertical part well developed and with horizontal part shallow. Temples well developed behind eyes; occiput smooth, deeply concave. Foramen magnum capitis well developed, surrounded by a deep fossa, distance between foramen and occipital carina c. 1.5 times its diameter. Postgena covered with vertical folds. Postgenal bridge smooth. Hypostomal folds present. Median sulcus of the postgenal bridge present. Inner hypostomal carina well developed, more distinct that outer hypostomal carina. Maxillo-labial complex with stipes, prementum, maxillary and labial palpi visible. Subgenal process weakly developed. Hypostomal area narrow. Hypostomal tooth not visible.

Mesosoma (Figs 1a; 1b) length 1.2–1.4 times width in female (1.3 ± 0.05, n = 60), 1.3–1.5 times width in male (1.4 ± 0.04, n = 22). Dorsal margin of mesosoma weakly convex in lateral view.

Transverse pronotal carina absent, pronotal shoulders strongly developed, rounded anteriorly. Vertical epomial carina present; horizontal epomial carina present (Figs 2a; 2b). Cervical pronotal area oblique, largely hidden in dorsal view. Lateral pronotal area broad, weakly concave. Netrion present (net Figs 2a; 2b), broad, approximately triangular, open ventrally, with foveolate sculpture.

Mesoscutum (Figs 1a; 1b), weakly convex, 2.1–2.8 times as long as scutellum, (2.4 ± 0.2, n = 60). Skaphion absent. Admedian lines absent. Notauli present, percurrent, usually deeply incised, crenulate. Notauli converging, closely approximated posteriorly, slightly dilated posteriorly. Humeral and suprahumeral sulci crenulate, but indistinct. Parapsidal lines present. Parascutal carina distinct. Mesoscutum foveolate. Transscutal articulation deep, crenulate. Mesoscutellum transverse,width 1.9–2.4 times length, (2.1 ± 0.13, n = 60); weakly convex, unarmed, posterior rim crenulate, sculpture like mesoscutum; length 3.3–8.0 times length of metascutellum in female (5.0 ± 0.75, n = 60), 3.8–5.7 times length of metascutellum in male (5.0 ± 0.6, n = 22). Metascutellum produced into a distinct rectangular plate, 4.0–8.0 times wider than long in female (5.0 ± 0.8, n = 60), 3.2–5.3 times wider than long in male (4.7 ± 0.6, n = 22).

Mesopleuron (Figs 2a; 2c; 2d) almost glabrous, with some scattered hairs. Speculum visible above the femoral depression, with a variable number of transverse ridges. Femoral depression large, deep, shining or with very smooth sculpture. Pleural pit distinct. Mesopleural carina indistinct. Posterodorsal corner of mesopleuron obtuse. Posterior mesepimeral area broad and shining. Sternaulus indistinct.

Propodeum (Figs 1a; 1b) in dorsal view, reduced and deeply excavate medially, lateral propodeal carinae separate the lateral propodeal areas from the deep and large metasomal depression which accommodates the horn of T1. The antero-dorsal ends of the carinae extend over the dorsal margin of the propodeum to form a projection.

Metapleuron entirely sculptured, divided by metapleural sulcus into a small dorsal area and in a large ventral area (Fig. 2a).

**Figure 4.**
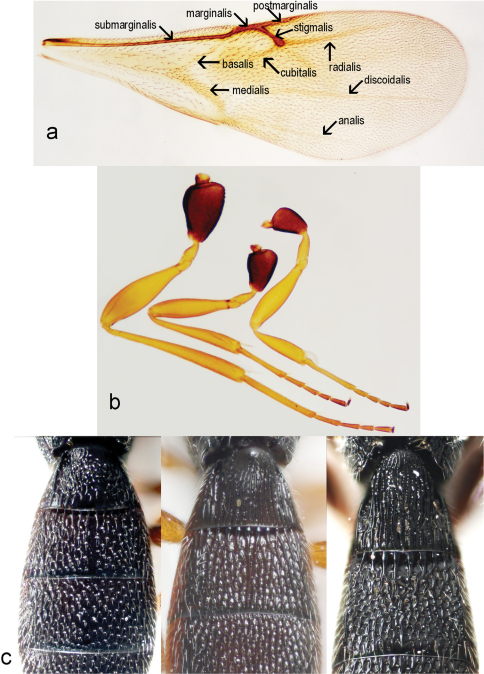
*Triteleia peyerimhoffi*: **a** – fore wing **b** – legs **c** – variability of sculpture of first 2 terga.

Macropterous, fore wings variable in length, not reaching apex of metasoma. Fore wing (Fig. 4a) covered with dense, short microtrichia. Length of fore wing 2.7–3.3 times width in female (3.0 ± 0.14, n = 60), 2.8–3.1 times width in male (2.9 ± 0.09, n = 22), 1.13– 1.5 times length of hind wing in female (1.3 ± 0.05, n = 60), 1.3–1.4 times length of hind wing in male (1.3 ± 0.04, n = 22), 2.7–3.3 times width of mesosoma in female (3.0 ± 0.11, n = 60), 2.7–3.1 times width of mesosoma in male (2.9 ± 0.1, n = 22). Fore wings with tubular submarginal, marginal, postmarginal and stigmal veins and with nebulous medial, cubital, anal, basal, discoidal and radial veins (Fig. 4a). Length of postmarginal vein 0.91–2.9 times length of marginal vein in female (1.3 ± 0.3, n = 60), 1.0–1.5 times length of marginal vein in male (1.2 ± 0.2, n = 22). Marginal vein length 0.7–1.3 times length of stigmal vein in female (1.03 ± 0.11, n = 60), 0.9–1.3 times length of stigmal vein in male (1.1 ± 0.09, n = 22).

Hind wing 4.0–6.1 times as long as wide in female (4.8 ± 0.4, n = 60), 4.4–5.7 times as long as wide in male (4.8 ± 0.3, n = 22), with three hamuli and complete submarginal vein. Marginal fringe short, width of hind wing 7.6 time length of marginal fringe.

Trochantellus present on all legs, tibial spur formula 1-1-1. The middle leg is the shortest (Fig. 4b).

Metasoma (Figs 1a; 1b) broadly sessile, depressed, in male with seven terga and seven sterna, in female with six terga, six sterna visible externally, homonomously segmented, T2–T4 subequal in length, T3 slightly the longest. Laterotergites well developed, narrow. Length of metasoma 2.0–2.6 (2.2 ± 0.13, n = 60) times length of mesosoma, 2.6–3.9 times width in female (3.2 ± 0.2, n = 60), 2.7–3.5 times width in male (3.0 ± 0.2, n = 22).

T1 with anterior margin carinate (especially visible in male), sublaterally with shallow depressions, with horn in female usually longitudinally costate. The apex of horn can be smooth, almost shining, or with longitudinally costae or with areolate rugulae (Fig. 4c). Length of T1 1.0–1.3 times its minimum width in female (1.1 ± 0.07, n = 60), 0.8–1.1 times its minimum width in male (1.0 ± 0.06, n = 22). Ratio between maximum and minimum width of T1 is 1.3–1.7 in female (1.5 ± 0.08, n = 60) and 1.3–1.5 in male (1.4 ± 0.05, n = 22).

Length of T2, 0.9–1.4 times the length of T1 in female (1.05 ± 0.06, n = 60) and 1.1–1.4 times the length of T1 in male (1.2 ± 0.07, n = 22). Maximum width of T2 1.4–2.0 its length in female (1.7 ± 0.1, n = 60) and 1.3–1.8 its length in male (1.6 ± 0.1, n = 22). Ratio between maximum and minimum width of T2 1.0–1.4 in female (1.3 ± 0.05, n = 60) and 1.2–1.4 in male (1.3 ± 0.04, n = 22). T3 is slightly the longest metasomal tergite, T3 length 1.0–1.3 times length of T2 in female (1.1 ± 0.05, n = 60), 1.0–1.2 times length of T2 in male (1.1 ± 0.04, n = 22) and 1.0–1.25 times length of T4 in female (1.1 ± 0.06), 1.0–1.2 times length of T4 in male (1.1 ± 0.04, n = 22). Maximum width of T3 1.3–1.8 times length in female (1.5 ± 0.11, n = 60), 1.3–1.6 times length in male (1.5 ± 0.1, n = 22). Ratio between maximum and minimum width of T3 is 1.0–1.1 in female (1.0 ± 0.01, n = 60) and 1.0–1.1 in male (1.0 ± 0.02, n = 22). Length of T4 1.2–1.6 times length of T5 in female (1.4 ± 0.07, n = 60), 1.3–1.6 times length of T5 in male (1.5 ± 0.08, n = 22) and length of T5 0.94–1.5 times length of T6 in female (1.2 ± 0.1, n = 60) and 1.7–3.3 times length of T6 in male (2.1 ± 0.4, n = 22). Ratio between maximum and minimum width of T4 is 1.2–1.4 in female (1.3 ± 0.05, n = 60), 1.1–1.3 in male (1.2 ± 0.04, n = 22) and ratio between maximum and minimum width of T5 is 1.2–2.1 in female (1.8 ± 0.13, n = 60) and 1.4–1.7 in male (1.5 ± 0.07, n = 22). Length of T6 0.6–1.2 times its maximum width in female (0.9 ± 0.01, n = 60) and 0.3–0.5 times its maximum width in male (0.4 ± 0.05, n = 22).

**Figure 5.**
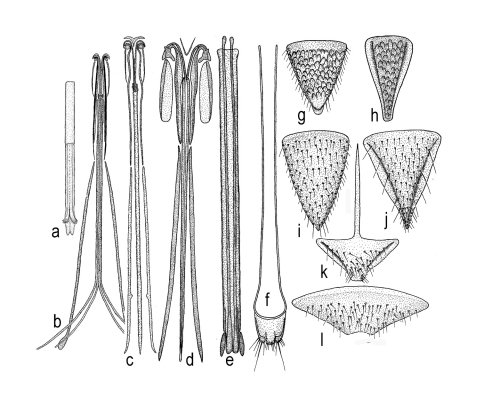
**a** – aedeagus in *Triteleia peyerimhoffi* **b** – ovipositor assembly in *Triteleia peyerimhoffi* **c** – ovipositor assembly in *Triteleia* sp. **d** – ovipositor assembly in *Apegus* sp. **e** – telescopic tube with lateral apodemes, incorporated into wall in *Triteleia* sp. **f** – T7, cerci and lateral apodeme in *Apegus* sp. **g** – T6 in female of *Triteleia peyerimhoffi* **h** – S6 in female of *Triteleia peyerimhoffi* **i** – T6 in female of *Triteleia* sp. **j** – S6 in female of *Triteleia* sp. **k** – S6 in female of *Apegus* sp. **l** – T6 in female of *Apegus* sp.

Ovipositor *Scelio*–type (Fig. 5b); the relation between ovipositor assembly length and metasoma length is shown in Fig. 2e.

Ovipositor assembly, very tiny, elongate. Proximal arms slender, short, 0.11 times length of ovipositor assembly; second gonapophyses assembly complex; gonoplacs elongate, 0.62 times ovipositor length; second gonocoxa 0.54 times gonoplac length. Gonoplacs weakly spatulate apically. First gonapophyses apically sharp. We cannot identify the proximal part of ventral membranous plate present in other *Triteleia* (Fig. 5c).

Lateral apodemes present, incorporated into wall of telescopic tube (Fig. 5e). Telescopic tube membranous with three or four sections. S6 without medial apodeme (Figs 5h; 5j).

Structure of ovipositor in *Triteleia dubia*, shows this species was misplaced in *Apegus* by Kieffer, because in *Apegus*, the ovipositor has a completely different structure, being *Ceratobaeus*–type (Fig. 5d).

The aedeagus (Fig. 5a) has two parts: the basal ring and aedeago-volsellar shaft. The basal ring is well developed, and represents 0.4 of copulatory organ length and 0.7 of aedeago-volsellar shaft. The aedeago–volsellar shaft has two aedeagal apodemes and two digiti volsellares. Each digitus has a row of five pits, each with a short tooth. The digiti, teeth and aedeagal apodemes are darker, more sclerotized than the rest of the copulatory organ.

#### Biology.

**Figure 6.**
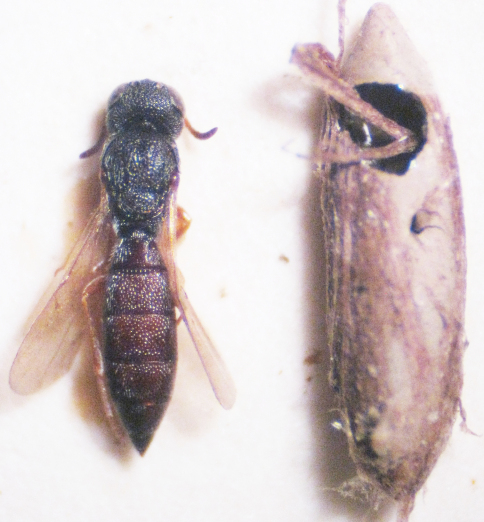
A female of *Triteleia peyerimhoffi* and its egg – host belongs to *Platycleis* *albopunctata.*

*Triteleia peyerimhoffi* is the third member in a tritrophic system, the other two being the plant-hosts and the orthopteran-host. We examined specimens of *Triteleia peyerimhoffi* obtained from the following plants: *Asphodelus* sp. (Asphodelaceae); *Ferula* sp., *Magydaris tomentosa* (both Apiaceae), *Tilia* sp. (Malvaceae) and *Quercus* sp. (Fagaceae). In most cases, *Triteleia peyerimhoffi* was obtained from the tettigoniids *Uromenus brevicollis insularis* or *Platycleis albopunctata* (Fig. 6). The relationship between *Asphodelus ramosum* – *Uromenus brevicollis insularis* – *Triteleia peyerimhoffi* (under the name *Triteleia dubia*) was previously noted by Cavalcaselle (1968) and the relationship between *Uromenus* *brevicollis* – *Triteleia peyerimhoffi*, was noted by Silvestri (1939) and Petit *et al*. (2007). *Triteleia peyerimhoffi* was collected from the end of June until the first part of October, with peak numbers in August.

#### Taxonomic comments.

Kieffer did not appreciate the variability of this species since he described the female of this species in 1906 in *Caloteleia*; males two years later in *Apegus*; and the male and female again (1910b) as *Ceratoteleia lugens*. According to Kieffer (1910a) *Ceratoteleia* and *Apegus* are very close, differing in details of the female clava and first metasomal tergite. We did not find the type specimens of these species. It seems that the types of *Ceratoteleia peyerimhoffi* and *Parapegus dubius* are lost. We looked for them in the collections of BMNH (Popovici and Notton), MNHN (collections of Jean-Jacques Kieffer and Paul de Peyerimhoff de Fontenelle) (Dr. Masner, Dr. Fusu & Dr. Claire Villemant) and HNHM (Dr. Sandor Csősz) but without success. It is possible that the type specimens of *Ceratoteleia lugens* are in the Naturhistorisches Museum, Vienna or in the Museo Civico di Storia Naturale di Trieste, but we have not visited these collections (Dr. Dominique Zimmermann and Dr. Fusu looked for this species in the Naturhistorisches Museum, but without success). When describing the male of *Ceratoteleia lugens*, Kieffer mentioned: ‘*chez le mâle, tous les tergites sont transversaux, le 6^e^ porte de chaque côté de son bord postérieur un petit appendice*‘. The bidentate or bispinose last tergite is a character state confirming that this species belongs to *Triteleia*. This species was obtained from *Foeniculum* sp. (Apiaceae); the host plant of the tettigoniids which *Triteleia peyerimhoffi* is known to attack. From Kieffer’s original descriptions it is impossible to find reliable characters to separate *Ceratoteleia peyerimhoffi* from *Ceratoteleia lugens* and from *Parapegus dubius*.

To clarify the taxonomic status of these species (ICZN, article 75.3.1), we here designate neotypes for *Parapegus* *dubius* and *Ceratoteleia peyerimhoffi*. We consider that the types of these species have been lost or destroyed: since we were unable to locate them in BMNH, MNHN, HNHM or in the Naturhistorisches Museum, Vienna. For *Parapegus dubius* we designate as neotype one female labeled: Hungary, Veröce 47°49.58'N, 19°1,30'E, 122m, 2–18.ix.2005, leg. Z. Nyiro (Malaise trap, CNCI). For *Ceratoteleia peyerimhoffi* we only have one male from Algeria. Because we have many specimens from Italy from the same host as the type specimens and because the male from Algeria is very similar to males from Italy, we decided to designate as a neotype a female of *Ceratoteleia peyerimhoffi* labeled: Italy, Guspini, 3.VIII.1933 (reared from *Asphodelus*). This neotype will be deposited in BMNH.

Kononova and Petrov (2000) described a new Palaearctic species of *Triteleia* from southeast Bulgaria, *Triteleia striolata*, based on two females, adding Israel to the distribution in 2008. Based on the description of its sculpture, ratios between sclerites, emergence dates, distribution and examination of pictures of the habitus, antenna and forewings of the holotype, we conclude that *Triteleia striolata* is a junior synonym of *Triteleia peyerimhoffi*.

Among the Palaearctic species described by Kieffer in *Ceratoteleia* there is one further species that has an uncertain status: *Ceratoteleia mediterranea*. Currently it is placed in *Calliscelio* (Johnson 1992; Kononova and Kozlov 2008), but we are convinced it is a *Triteleia*, and possibly another junior synonym of *Triteleia peyerimhoffi*. We have not found the type specimens, although the senior author and Dr. Masner saw two females in MNHN identified by Maneval as *Caloteleia mediterranea* and the senior author saw a similar specimen in FBIN also identified as *Caloteleia mediterranea*. The main difference between these specimens and *Triteleia peyerimhoffi* is the overall size and the ratio between the length and maximum width of the metasoma. It is possible these specimens are extreme examples of *Triteleia peyerimhoffi*, but until we see more specimens we prefer to not include these specimens in *Triteleia peyerimhoffi*.

#### Material examined.

**FRANCE: 17 females,** Lot Escamps, 5–31.viii.1995, Malaise trap, leg. H. Tussac (CNCI); **1 male,** Lot Escamps, 5–31.viii.1995, Malaise trap, leg. H. Tussac (CNCI); **1 female,** Dordogne, Couze St. Front, 27.vi–11.vii.1993, Malaise trap, leg. H. Tussac (CNCI); **2 females,** Dordogne, Couze St. Front, 1.ix.1994–22.ii.1995, Malaise trap, leg. J. N. Revol (CNCI); **1 female,** Gard, St. Félix de Paulliéres, La Hourne Haute, 7–14.vii.1996, Malaise trap, leg. J. F. Vayssiéres (CNCI); **2 females**, Bouches-du-Rhône, Fonscolombe, 17.vii.1990, leg. M. de V. Graham (BMNH(E)1995-489); **1 male**, Bouches-du-Rhône, nr. Rognes, 16.vii.1979 (BMNH(E)1995-489); **1 female**, Bouches-du-Rhône, Fonscolombe, 25.vii.1990, leg. M. de V. Graham (BMNH(E)1995-489); **1 female**, Bouches-du-Rhône, Fonscolombe, 4.viii.1986, leg. M. de V. Graham (BMNH(E)1995-489); **1 female**, Bouches-du-Rhône, Fonscolombe, 15.viii.1980, ex. *Caloteleia coriaria* (Fabaceae) gall, leg. M. de V. Graham (BMNH(E)1995-489); **1 female**, Bouches-du-Rhône, Fonscolombe, 29.vii.1979, leg. M. de V. Graham (BMNH(E)1995-489); **1 female**, Pignans, 4.ix.1965, leg. J. Barbier (MNHN, 7237); **1 female**, Esbarres, C. D'OR, 6.viii.1955, leg. J. Barbier (MNHN, I536)

**HUNGARY: 3 females,** Veröce, 47°49.58'N, 19°1.30'E, 122m, 2–18.ix.2005, Malaise trap, leg. Z. Nyiro (CNCI).

**ITALY: 3 females,** Bienca, 20.ix–19.x.1985, leg. A. Casale (CNCI); **2 females,** Toscana Sesto Fior. ix.1943, leg. L. Ceresa (OPPC); **1 male & 2 females**, Sardegna, Macomer, 8.vii.1957, reared from *Uromenus brevicollis* *insularis* on *Ferula* sp. (FBIN); **3 males** & **3 females**, Sassari, Bunnari, 8.vii.1957, reared from *Uromenus brevicollis* *insularis* on *Magydaris tomentosa* (FBIN); **13 males & 52 females**, Guspini, vii.1934, reared from *Asphodelus* (FBIN); **24 males & 114 females**, Guspini, 7.vii.1934, reared from *Asphodelus* (FBIN); **24 males & 1 female**, Guspini, vi–vii.1933, reared from *Asphodelus* (FBIN); **11 males & 79 females**, Guspini, 19.vii.1934, reared from *Asphodelus* (FBIN); **67 males & 38 females**, Sessa Aurunca, 7.vii.1934, reared from *Asphodelus* (FBIN); **2 females & 4 males**, Matera, 1934, reared from *Platycleis grisea* (FBIN); **1 male & 7 females**, ?locality, 1964, reared from *Uromenus brevicollis* *insularis*, leg. Crovetti (FBIN); **189 males & 84 females**, Guspini, vi.1934, reared from *Asphodelus* (FBIN); **1 female & 1 male**, Toscana Sesto Fior. vii.1943, leg. L. Ceresa (FBIN); **25 males & 13 females**, Mandas vii.1933, reared from *Asphodelus* (OPPC); **5 females**, Guspini, vii.1933, reared from *Asphodelus* (FBIN); **2 males & 1 female**, Guspini, 4.viii.1933 (OPPC); **2 females**, Guspini, 3.viii.1933, reared from *Asphodelus* (OPPC); **1 female**, Caprioli, 21.vii.1936 (OPPC); **2 males & 1 female**, Nuoro, 13.vii.1933, reared from *Asphodelus* (OPPC); **1 female**, Sessa Aurunca 27.vii.1934 (OPPC); **6 males**, ?locality, viii.1933, reared from eggs of Orthoptera, leg. Dr. Provasoli (OPPC).

**CROATIA: 1 female,** Krk Isle 24.viii.2007, swept, leg. M. Mitroiu (OPPC).

**ROMANIA: 1 female,** Bârnova forest, N46°59'37.0", E27°35'27.1", 8.ix.2004, swept, leg. O. Popovici (OPPC); **1 female,** Bârnova forest, N46°59'37.0", E27°35'27.1", 12.viii.2010, Malaise trap, leg. M. Popovici (OPPC); **2 females,** Bârnova forest, 27.iii.2006, obtained from dead wood of *Tilia* sp., leg. L. Fusu & M. Dascălu, (OPPC); **1 female,** Mârzeşti forest, 14.ii.2006, from dead wood of *Quercus* sp., leg. L. Fusu & M. Dascălu (OPPC).

**GREECE**: **1 female &** **1 male,** Krousia Mts., N41°11'32,4", E23°03'59,5", 18–24.vii.2007, Malaise trap, leg. G. Ramel (OPPC); **1 female,** Krousia Mts., N41°11'32,4", E23°03'59,5", 8–14.viii.2007, Malaise trap, leg. G. Ramel (OPPC); **1 female,** Promohonas site, N41°22'25.32", E23°22'18.84", 11–17.vii.2007, Malaise trap, leg. G. Ramel (OPPC); **2 females,** Midway site, N41°18'49.8", E23°16'35,6", 14–21.vii.2008, Malaise trap, leg. G. Ramel (OPPC); **1 male &** **1 female,** Midway site, N41°18'49.8", E23°16'35.6", 21– 7.vii.2008, Malaise trap, leg. G. Ramel (OPPC), **1 female,** Midway site, N41°18'49.8", E23°16'35.6", 8–14.ix.2008, Malaise trap, leg. G. Ramel (OPPC); **1 male,** Midway site, N41°18'49.8", E23°16'35.6", 28.vii–3.viii.2008, Malaise trap, leg. G. Ramel (OPPC); **2 females &** **1 male,** Thessalia, Kalambaka, 14–20.viii.1979, hillside meadow, leg. M. C. Day, G. R. Else & D. Morgan (BMNH(E)1979-312).

**SPAIN:** **1 male**, Andalucía, Jaén, Santa Elena, 5.vii.1974, leg. Z. Bouček (BMNH(E)1974-321).

**PORTUGAL: 1 male**, Madeira, pre-1855, leg. Wollaston (BMNH(E)1855-7,).

**ALGERIA:** **1 male**, Oran, Douar belbaid, reared from *Asphodelus,* leg. J. Barbier (6565 MNHN).

**JORDAN: 1 male,** NW corner, c. 16 km WWN Aljun, 21.v.2007,

32°27.074'N, 35°42.404'E, 600m, leg. J. Bezdek (CNCI).

## Discussion

*Triteleia peyerimhoffi* is a widely distributed species in southern Europe and because of this wide distribution it appears to form local races which appear different. Therefore, it is easy for researchers working with a limited series of specimens from a limited geographic region to misinterpret this intraspecific variability as representing additional species. Indeed, specimens from the extremes of the species’ distribution may look like different species; however, studying a large number of specimens shows an almost continuous morphological gradation between these extremes.

In other parasitic Hymenoptera PCA was successfully used to separate closely related species (Popovici and Buhl 2010; Fusu 2010; Polaszek 2004; Bernardo et al. 2008). We used PCA here to see if, and how morphology of this species varied with distribution.

**Figure 7.**
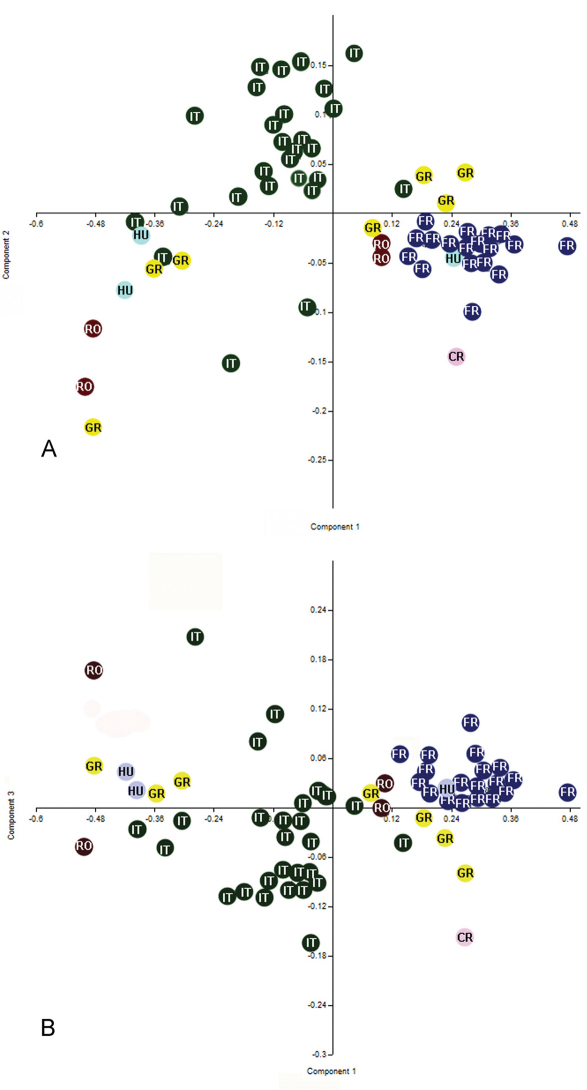
Scatter plots of the first three factors from the analysis of the log–transformated data for some population belongs to *Triteleia peyerimhoffi* (A – PC1 and PC2; B– PC1 and PC3); RO– Romania, IT– Italy, GR– Greece, FR– France, CR– Croatia, HU– Hungary.

The first six principal components of PCA explain 80.34% of the total variance (one indicated by Jolliffe cut-off value = 0.002; eigenvalues of the first six PCs > 0.002). 95% bootstrapped confidence intervals are given for the eigenvalues (Table 1). The first three principal components of this analysis are plotted in Figures 7a and 7b; they explain 67.35% (PC1), 6.99% (PC2) and 6% (PC3) respectively of the total variance. Both graphs of PC1 on PC2 and PC1 on PC3 displayed a trend of separation between specimens (Figs 7a, 7b). The contributions of variables in this dispersion are showed in the Table 2.

Because in both the graph of PC1 on PC2 and PC1 on PC3, but especially in PC1 on PC2 we obtained a relatively clear separation between the specimens from France and specimens from Italy, we wanted to see if there is a significant difference between these two populations.

One-way ANOVA of all variables indicated significant differences between populations for all variables excepting one (LT6 min: F= 1.865, p= 0.13). The analysis proceeded by pair-wise comparisons (post-hoc) using the Games Howel test after the Levene test revealed unequal variances (p <0.05). The Games Howel test indicated small but significant differences between populations for most variables (p<0.05) (Table 3). The main differences appear between specimens from Italy and specimens from France, in this case almost all characters showed significant differences. Also there were a few significant differences between specimens from France and specimens from Greece (for six characters), between specimens from France and specimens from Hungary (for one character) and between specimens from France and specimens from Romania (for one character). There were no significant differences between specimens from France and the specimen from Croatia. Interestingly there weren’t any significant differences between specimens from Italy and specimens from Greece, Croatia, Romania and Hungary. Also, there were no significant differences between specimens from Greece, Romania, Croatia and Hungary. These results demonstrate that all these specimens from different populations belong to the same species, specimens from France and specimens from Italy being at different extremes with populations from Greece, Croatia, Romania and Hungary, lying between these two populations.

Because characters of the metasoma were very important in the PCA analysis we decided to analyze the size and shape of the metasoma in *Triteleia peyerimhoffi* to reveal more detail of the variability within this species.

**Figure 8.**
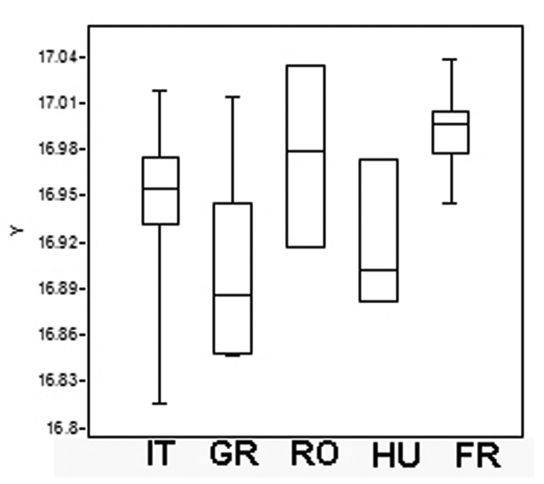
Boxplot of metasoma Log– Transformed Centroid Size (Log–CS) screening the metasoma size variation in *Triteleia peyerimhoffi* populations. (IT– Italy, GR– Greece, RO– Romania, HU– Hungary, FR– France).

One way ANOVA found significant differences in centroid size between populations (F= 7.17; P = 0.001). The distribution of the log-transformed centroid size is shown in Fig. 8. Levene’s test revealed a normal distribution of all populations (*p*>0.05) and Tukey’s pairwise mean comparisons showed a significant difference between three of them (Table 4). Again, in the analysis of the metasoma, there were some significant differences between France and Italy, Greece and Romania and between France and Greece. There were no differences however between Hungary and Romania or between Hungary and Greece, no difference between Italy and Romania or between Italy and Greece, no difference between France and Romania, or between specimens from France and Hungary or Croatia (Table 4). We conclude, as before, that although there are some significant differences between some populations, overall the specimens belong to the same species.

**Figure 9.**
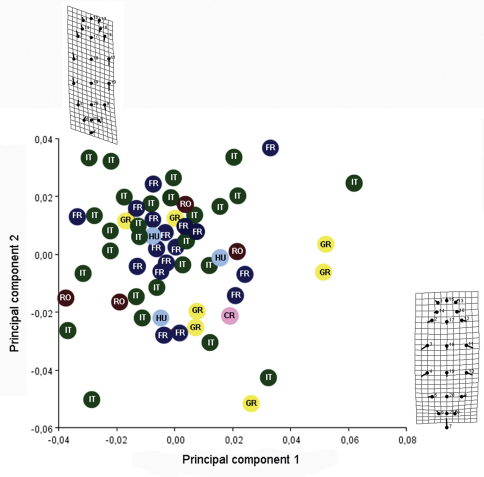
PC1 vs PC2 screen plot and thin plate deformation grids show the transformation of shape of metasoma along the two axes. (RO– Romania, IT– Italy, GR– Greece, FR– France, CR– Croatia, HU– Hungary).

**Figure 10.**
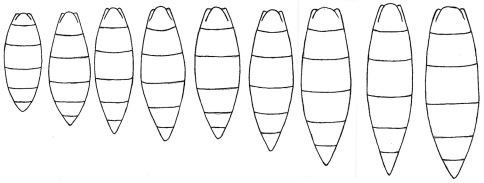
Morphological gradation between the shortest metasoma and the longest metasoma in *Triteleia peyerimhoffi*.

The result of analysis of the shape of the metasoma is shown on the plot of principal components. The first two principal components are shown in Figure 9. PC1 accounts for 35.21% of the shape variability and PC2 explains 31.38% of the variability. Thin plate deformation grids show the transformation of shape along the two axes.The MANOVA permutation test found insignificant overall difference between shapes (p>0.05) of metasoma in the six examined populations. The result of this test, confirm our view that all populations belong to the same species. So, in Fig. 9 it is impossible to separate specimens from their provenance, because there is a mixture of specimens belonging to the different populations. Hence we consider this variation in size and shape of the metasoma to be intraspecific variability. Furthermore, we found an almost continuous gradation between the shortest and the longest metasoma (Fig. 10).

Multiple regression of shape on size was performed. The results revealed that only 10.88% of variability of shape is predicted by size (Permutation test against the null hypothesis of independence/ Number of randomization rounds: 10000, P-value: <0.001).

**Figure 11.**
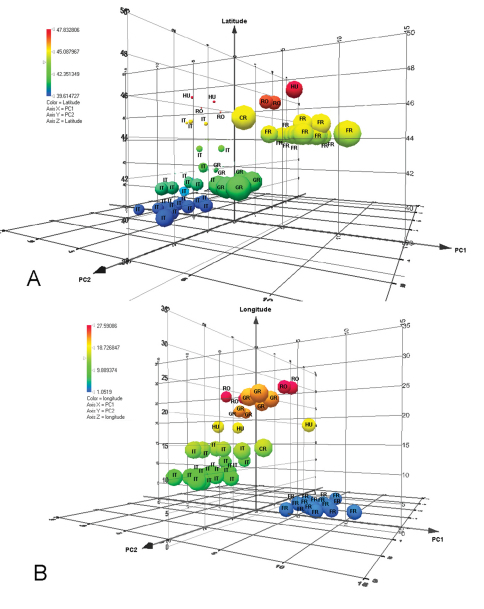
3D–plot of distribution of data according to PC1 and PC 2 and A: latitude; B: longitude. **(**RO– Romania, IT– Italy, GR– Greece, FR– France, CR– Croatia, HU– Hungary).

We therefore assert that the great variability of this species to geographical variation. The correlation of geography with these variables was shown by Spearman’s rank coefficient. There was a significant correlation between longitude and PC1 (Spearman’s correlation = - 0.52; p<0.01) and latitude and PC2 (Spearman’s correlation = - 0.62; p<0.01). Therefore, in the case of this species, there is a correlation between longitude and LA3, LT5, width of metascutellum, LT4, LE and LT6 and also, between latitude and minimum width of T6, length of T6, Lt, length of T3, length of T4, length of T2 and length of T5. The relation between latitude and longitude on PC1 and PC2 can be seen in Fig. 11A & B. So, the gap between populations on Fig. 7A and 7B can be explained as a gap between the sites where the specimens were collected. The majority of specimens from Italy were collected from Sardinia and just three from continental Italy. Similarly for the other material: from Greece we analyzed specimens from northern Greece only; from Romania, only specimens from the North-East; from France, only specimens from a limited area. Hence there are many unrepresented areas between the sampled populations. Consequently we think this is the reason for the gap between the populations from France and Italy. Also, we have few specimens from northern Italy, and this population is separated from that in France by the Western Alps.

A large variation in size, shape and sculpture of the metasoma is not something that is unusual within scelionid species, e.g., Vecher (1980) notes the intraspecific variability of the metasoma in *Telenomus angustatus* reared from tabanid eggs. He analysed 5274 females and 1009 males and noted great variability in the shape and size of the metasoma and in the sculpture of T1 and especially T2. The most interesting thing is that variability was observed in specimens which were the progeny of thelytokous females. He confirmed his conclusion that all specimens belonged to the same species with allozyme electrophoresis.

Within the Chalcidoidea, Popescu (2004) found a great variability in the size and shape of antennomeres in females of *Idiomacromerus pallistigmus* Askew and *Eridontomerus arrabonicus* Erdös (both Torymidae). The specimens were reared as from *Blascoa ephedrae* Askew (Pteromalidae) from *Ephedra* *distachya* L. (Ephedraceae) and *Tetramesa scheppigi* (Schlechtendal) (Eurytomidae) from *Stipa lessingiana* (Poaceae) respectively.

Kononova (2001) gave great importance to the influence of hosts in the morphology of parasitoids. She wrote: ‘adaptation of scelionids to hosts from more advanced orders cause sharp modifications of not only the habitus (strongly shortened body), but also some other morphological characters (e.g. shortening of the abdomen through reduction of its apical segments)’.

The influence of hosts on the morphology of parasitoids was demonstrated by Johnson et al. (1987). They studied the intraspecific variability in *Telenomus alsophilae* reared from different hosts in the laboratory under controlled conditions and emphasised the strong influence of hosts in the morphology of the antennae of their parasitoids. Another interesting fact shown by Johnson et al. (1987) was that specimens reared from field collected eggs showed substantially greater coefficients of variation than the laboratory-reared specimens most likely as a result of uncontrolled environmental variables.

It is very probable that *Triteleia peyerimhoffi* has not just a single host, but uses a number of similar tettigoniid hosts and differences between the size and shape of the eggs of different host species, and differences between the size and shape of eggs within the same host species under different environmental conditions are a source of intraspecific variability within and between parasitoid populations.

We conclude that *Triteleia peyerimhoffi* is a species with a wide circum-Mediterranean distribution and is one of the most important eggs parasitoids of *Uromenus brevicollis*. As an alternative host it uses *Platycleis albopunctata* which explains its presence in areas (e.g. Romania) where there is no *Uromenus*, but where *Platycleis albopunctata* common (Iorgu I., pers. comm*.*).

**Table 1.** Principal Components and the bootstrapped confidence intervals.

PC	Eigenvalue	% variance	Eig 2.5%	Eig 97.5%
1	0.063821	67.351	61.114	74.243
2	0.00663	6.997	4.5251	10.308
3	0.005683	5.9973	3.2998	10.638
4	0.004301	4.5388	2.7452	72.321
5	0.002332	2.4606	1.1542	41.235
6	0.002188	2.3087	1.2735	33.399

**Table 2.** The contribution of the variables along the first three principal components

Axis	Loading	Variables
PC1	0.2951	LA3
	0.2375	LT5
	0.2348	width of metascutellum
	0.209	LT4
	0.2032	LE
	0.2024	LT6
PC2	0.7473	minimum width of T6
	0.2352	length of T6
	0.2321	Lt
	0.1575	length of T3
	0.1565	length of T4
	0.1146	length of T2
	0.09395	length of T5
PC3	0.4948	minimum width of T6

**Table 3.** Results of Multiple Comparisons: Games – Howell test (the Table includes only variables and populations with only significant differences)

Variables	Populations		Mean Difference	Std. Error	p- value	95% Confidence Interval	
						Lower Bound	Upper Bound
LH	France	Italy	0.04	0.01	0.000	0.02	0.05
WH	France	Italy	0.08	0.01	0.000	0.06	0.09
WH	France	Greece	0.06	0.02	0.045	0.00	0.12
POL	France	Italy	0.05	0.01	0.000	0.03	0.08
distance between eyes at level of anterior ocellus	France	Italy	0.06	0.01	0.000	0.05	0.08
Hfd	France	Italy	0.08	0.01	0.000	0.06	0.11
distance between compound eye and frontal depression	Romania	France	-0.08	0.02	0.031	-0.16	-0.01
distance between compound eye and frontal depression	France	Italy	0.06	0.02	0.027	0.01	0.12
HE	France	Italy	0.09	0.00	0.000	0.07	0.10
LE	France	Italy	0.09	0.01	0.000	0.07	0.11
gen	France	Italy	0.08	0.01	0.000	0.04	0.11
width of mesosoma	France	Italy	0.07	0.01	0.000	0.05	0.09
length of mesoscutum	France	Italy	0.07	0.01	0.000	0.05	0.08
Wscut	France	Italy	0.08	0.01	0.000	0.06	0.09
width of metascutellum	France	Italy	0.10	0.01	0.000	0.07	0.12
length of T1	France	Italy	0.05	0.01	0.000	0.03	0.08
minimum width of T1	France	Italy	0.08	0.01	0.000	0.06	0.10
maximum width of T1	France	Italy	0.06	0.01	0.000	0.05	0.08
maximum width of T1	France	Greece	0.06	0.02	0.040	0.00	0.11
length of T2	France	Italy	0.05	0.01	0.000	0.03	0.07
maximum width of T2	France	Italy	0.04	0.01	0.000	0.03	0.06
length of T3	France	Italy	0.03	0.01	0.030	0.00	0.05
maximum width of T3	France	Italy	0.06	0.01	0.000	0.04	0.07
length of T4	France	Italy	0.05	0.01	0.000	0.03	0.08
minimum width of T4	France	Italy	0.09	0.01	0.000	0.07	0.10
minimum width of T1	France	Greece	0.08	0.02	0.010	0.02	0.14
length of T5	France	Italy	0.07	0.01	0.000	0.04	0.10
minimum width of T5	France	Italy	0.08	0.01	0.000	0.05	0.10
minimum width of T5	France	Greece	0.11	0.02	0.002	0.05	0.16
minimum width of T6	France	Hungary	0.00	0.00	0.003	0.00	0.00
Lfw	France	Italy	0.07	0.00	0.000	0.06	0.09
Wfw	Greece	France	-0.05	0.01	0.041	-0.10	0.00
Wfw	France	Italy	0.06	0.00	0.000	0.05	0.07
length of marginal vein	France	Italy	0.08	0.01	0.000	0.05	0.12
Lhw	France	Italy	0.07	0.01	0.000	0.05	0.09
Whw	France	Italy	0.09	0.01	0.000	0.06	0.11
Whw	France	Greece	0.07	0.01	0.008	0.02	0.11

**Table 4.** Results of Multiple Comparisons: Tukey’s test (the table includes only populations with significant differences)

Populations		Mean Difference	Std. Error	P value	95% Confidence Interval	
Italy	France	-0.04	0.01	0.01	-0.08	-0.01
Greece	Romania	-0.08	0.03	0.03	-0.16	-0.01
France	Greece	0.09	0.02	0.00	0.04	0.15

## Supplementary Material

XML Treatment for Triteleia peyerimhoffi
